# Upper Hemi-Sternotomy Provides Benefit for Patients with Isolated or Combined Mitral Valve Surgery

**DOI:** 10.3390/medicina58020142

**Published:** 2022-01-18

**Authors:** Cenk Ulvi Oezpeker, Fabian Barbieri, Daniel Hoefer, Nikolaos Bonaros, Michael Grimm, Ludwig Mueller

**Affiliations:** 1 Department of Cardiac Surgery, Medical University of Innsbruck, 6020 Innsbruck, Austria; daniel.hoefer@tirol-kliniken.at (D.H.); nikolaos.bonaros@tirol-kliniken.at (N.B.); michael.grimm@tirol-kliniken.at (M.G.); ludwig.mueller@tirol-kliniken.at (L.M.); 2 Department of Cardiology, Medical University of Innsbruck, 6020 Innsbruck, Austria; fabian.barbieri@i-med.ac.at

**Keywords:** mitral valve surgery, contraindications for mini-thoracotomy, upper hemi-sternotomy, less invasive approach

## Abstract

*Background and objectives*: Certain clinical and anatomical conditions are absolute or relative contraindications for safe mitral valve surgery via the right mini-thoracotomy access. It is uncertain whether patients with these contraindications may benefit from the less invasive approach via upper hemi-sternotomy compared to standard full sternotomy. *Materials and methods*: Out of 2052 mitral valve surgery patients, operated from 6/04 through 2/19, 1535 were excluded due to the different criteria for eligibility to both approaches. Out of these, 350 received full sternotomy and 167 upper hemi-sternotomy. After propensity score matching, 164 pairs were analyzed for operative variables, postoperative complications and 30-day and one-year survival. *Results*: Upper hemi-sternotomy was associated with a survival benefit of 30 days (99.4% vs. 82.1%; *p* < 0.001) and one-year (93.9% vs. 79.9% *p* < 0.001, HR 0.26, 95% CI 0.14–0.49). Cardiopulmonary bypass and aortic cross-clamp times were comparable in both groups. Upper hemi-sternotomy resulted in less low cardiac output syndrome (18.9% vs. 31.1%; *p* = 0.011); ventilation time (8 vs. 13 h; *p* < 0.001), length of intensive care stay (1 vs. 2 days; *p* < 0.001) and total hospital stay (8 vs. 9 days; *p* < 0.001) were shorter in the upper hemi-sternotomy group. *Conclusion*: In patients undergoing mitral valve surgery, upper hemi-sternotomy is associated with short- and mid-term survival benefits as well as lower postoperative complication rates compared to full sternotomy. Hence, the less invasive upper hemi-sternotomy can be a valid approach in patients with contraindications for right mini-thoracotomy.

## 1. Introduction

In most dedicated centers the preferred access for isolated mitral valve surgery in patients with an acceptable risk profile is right mini-thoracotomy [1,2,3,4]. However, certain clinical and anatomical conditions are limitations for safe mitral valve surgery with this access. Concomitant cardiac surgery, other than tricuspid valve repair or atrial ablation procedures, severe right-sided pulmonary adhesions, significant mitral annulus calcification with the need for en-bloque decalcification, severely atherosclerotic aorta or femoral arteries, not being amenable for peripheral cannulation and a dilated ascending aorta >45 mm are regarded as contraindications for mini-thoracotomy access and/or femoral cannulation by most surgeons [3,5,6,7]. Further, relative contraindications (e.g., advanced age and severe pulmonary hypertension) are based on institutional experience and may additionally limit the number of patients suitable for this approach. In most centers, patients who are considered to be unsuitable candidates for mini-thoracotomy are operated on with a conventional full sternotomy.

Based on more than 20 years’ experience in minimally invasive cardiac procedures—including partial sternotomy and mini-thoracotomy for aortic valve replacement, and more than 1000 video-assisted or fully endoscopic mitral valve procedures via mini-thoracotomy [8]—we were inspired to seek an alternative and less invasive option for certain risk profile patients in order to close the gap between the smallest possible and full sternotomy access. It was therefore a logical step to implement upper hemi-sternotomy with the extended transseptal incision as a complementary less invasive access in mitral valve patients [9].

Upper hemi-sternotomy allows the use of standard surgical instruments with direct vision and central cannulation and the access is also suitable for combined procedures, particularly aortic valve replacement. Since the first description by Meyer et al. in 1965 [10], with access via the left atrial roof the extended transseptal approach has been established in several programs and different publications have underlined the safety of this method [9,11,12,13]. However, the few existing publications have not made clear if these patients with a certain risk profile would benefit from this approach compared to median sternotomy. Moreover, in all these studies, percutaneous cannulation of the femoral vein for the purpose of inferior vena cava drainage was not taken into consideration for this approach either.

Whereas the advantages of minimally invasive mitral valve surgery via mini-thoracotomy are well described [14,15,16,17], the advantages and disadvantages of the less invasive upper hemi-sternotomy in mitral valve patients are uncertain. It was therefore the purpose of this study to evaluate the short- and mid-term results of the upper hemi-sternotomy access for mitral valve surgery in patients not suitable for endoscopic surgery.

## 2. Material and Methods

### 2.1. Patients

**Figure 1:**
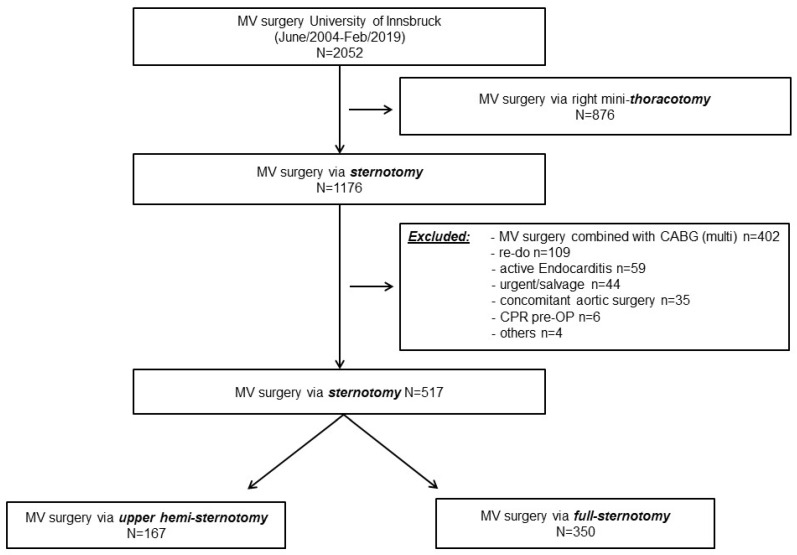
Mitral valve surgery flowchart.

This retrospective analysis includes 2052 patients who underwent mitral valve surgery at our center between June 2004 and February 2019 documented in the institutional database. Of these patients, 876 were operated on via right mini-thoracotomy and were thus excluded from the analysis, leaving 1176 patients who underwent mitral valve surgery via sternotomy (Figure 1). A total of 659 patients demonstrated exclusion criteria for eligibility to both approaches, i.e., concomitant coronary bypass grafting, redo surgery, active endocarditis, urgent or salvage surgery, concomitant surgery of the ascending aorta or preoperative resuscitation. Eight patients were excluded due to incomplete data, leaving 517 patients (full sternotomy *n* = 350, upper hemi-sternotomy *n* = 167) who were finally included in the data analysis.

Written informed consent for scientific use of clinical data was obtained from all patients as part of the quality assurance program of our department, approved by the local ethics committee (EC number: 1203/2019) and the Austrian Ministry of Health. The investigation conforms to the principles outlined in the Declaration of Helsinki.

### 2.2. Study Cohort

In case of a certain risk profile, mitral valve surgery was conducted via full sternotomy up to 2011, until the implementation of upper hemi-sternotomy access that year. To create a homogeneous cohort, these above-mentioned primary center-specific criteria were used as exclusion principles for our study population, making both groups eligible for either full or upper hemi-sternotomy. However, over the years the criteria changed and with rising institutional experience, a tailored approach for decision-making for mitral valve access was performed in the valve team. For analysis, patients were divided into two groups (Figure 1): group 1 consisted of patients who underwent full sternotomy and group 2 underwent mitral valve surgery via upper hemi-sternotomy.

Clinical data were obtained from the database of our mitral valve surgery program and from the hospital’s digital patient management system. Baseline characteristics included demographic parameters, the EuroSCORE II [18] and the New York Heart Association class (NYHA). In addition, concomitant diagnoses such as diabetes mellitus, peripheral vascular disease, chronic obstructive pulmonary disease, atrial fibrillation, history of cerebral stroke, renal failure requiring dialysis and history of acute cardiac decompensations within three months preoperatively were assessed (see Table 1). Further risk factors included pulmonary artery pressure, renal function and NT-proBNP. Moreover, we recorded mitral valve specific data such as etiology of mitral valve disease, mitral valve dysfunction according to Carpentier’s classification [19], mitral annulus calcification and left ventricular ejection fraction (LV-EF). Intra-, peri- and postoperative parameters were assessed for detailed analysis.

### 2.3. Surgical Procedures

The upper hemi-sternotomy approach, with extension of the transseptal incision into the left atrial roof, was described in detail by Gillinov and Svensson et al. [9,20]. Briefly, starting at the sternal notch the sternotomy was performed with extension into the left fourth intercostal space. Cannulation for cardiopulmonary bypass was performed via the ascending aorta; the superior vena cava was cannulated directly, while the inferior vena cava was cannulated percutaneously via the femoral vein using the Seldinger technique guided by transesophageal echocardiography and, if necessary, by C-arm fluoroscopy. Correct positioning was achieved after establishing cardiopulmonary bypass and pulling back the cannula into the orifice of the inferior vena cava. The superior and inferior vena cava were snared with vessel loops. To facilitate snaring of the IVC we used an aortic aneurysm clamp and Mersilene band.

After aortic cross-clamping with a rigid, right angled clamp, antegrade was followed by retrograde (by direct coronary sinus cannulation after opening of the right atrium); St Thomas’ II cardioplegia was administered for myocardial protection. The next step was incision of the interatrial septum at the level of the fossa ovalis, connecting to the right atrial incision and extending into the roof of the left atrium. The extended transseptal incision was performed approximately two centimeters away from the ascending aorta and the MV-annulus to leave enough myocardium for closure and not to distort the left main coronary artery. Transection of the sinus node artery was sometimes inevitable. Excellent exposure of the mitral valve was achieved using handheld peanut sponges mounted on a slightly curved clamp to retract the interatrial septum.

In case of full sternotomy, cannulation was similar with the exception of direct cannulation of inferior vena cava. Access to the mitral valve was performed via dissection of the interatrial groove.

Almost all reconstruction techniques such as leaflet resection with or without the use of bovine pericardium, chordal replacements, en-bloque decalcification with reconstruction of the annulus and a sliding atrium plasty were performed via both accesses.

Additional tricuspid and/or aortic valve interventions were performed after completion of the mitral valve procedure.

### 2.4. Outcome Parameters

The major outcome parameter was survival at 30 days and one-year. Further outcome parameters were mitral valve repair rates, number of conversions to full sternotomy, operative times (cardiopulmonary bypass and aortic cross-clamp times) and re-thoracotomy due to tamponade or excessive bleeding. Moreover, new onset kidney failure with renal replacement therapy and duration of postoperative mechanical ventilation were assessed. Further outcome parameters included postoperative cardiac low output syndrome, the number of red blood cell units transfused, perioperative myocardial infarction, the need for implantation of a permanent pacemaker, cerebral stroke, sepsis, deep sternal wound infection, pneumonia, multi-organ failure, length of intensive care unit and total hospital stay.

### 2.5. Statistical Analysis

Continuous variables are expressed as median and interquartile range; categorical variables are reported as number and percentage. Differences in continuous variables were compared using a *t* test or Mann–Whitney U test according to their distribution. Differences in categorical variables between the two groups were analyzed with the chi-square test.

To minimize the effect of a possible selection bias or confounding by other parameters due to a non-randomized group assignment, a propensity score matchmaking model was calculated. For this purpose, a binary logistic regression was conducted including presence of atrial fibrillation, etiology of mitral valve disease (primary or secondary), moderate to major annulus calcifications, EuroSCORE II and the type of surgery performed (isolated mitral valve surgery, mitral valve and tricuspid valve surgery, mitral valve and aortic valve surgery, mitral valve and aortic valve and tricuspid valve surgery) as these were regarded as the most important risk and confounding factors. Given probabilities were then used for a nearest neighbor matchmaking process (1:1) without replacement by using a matching tolerance of 0.01, which resulted in 164 pairs. Subsequently, Kaplan–Meier curves were plotted for univariate analysis of survival and differences were assessed by using the log-rank test. Statistical analysis was conducted using IBM SPSS, version 24 (IBM Corporation, Armonk, NY, USA); graphics were designed using GraphPad PRISM, version 5 (GraphPad Software, Inc., La Jolla, CA, USA). Additionally, *p* values < 0.05 were considered statistically significant.

## 3. Results

### 3.1. Baseline Characteristics

Baseline and intraoperative characteristics are listed in Table 1 and Table 2. Nearly all the given baseline criteria showed no statistical significant difference between the cohorts, either before or after the matchmaking process. After propensity score matching the systolic pulmonary arterial pressure was significantly lower in the upper hemi-sternotomy cohort (45 mmHg vs. 50 mmHg, *p* = 0.024). Moreover, the prevalence of arterial hypertension was higher (81.1% vs. 68.3%, *p* = 0.008) in the upper hemi-sternotomy cohort. In addition, the prevalence of hospitalization for acute heart failure before surgery was higher in the upper hemi-sternotomy cohort (12.8% vs. 5.5%, *p* = 0.022). However, preoperative NT-proBNP levels were similar between the groups.

Regarding etiology, there was almost no difference in the incidence of mitral valve pathology (*p* = 0.624). Identical results were found for moderate to severe annulus calcification (35.4% vs. 36%, *p* = 0.908).

There was no statistically significant difference in the numbers of isolated mitral surgery, mitral valve repairs vs. replacements or in the number of concomitant procedures such as aortic valve and/or tricuspid valve surgery between the cohorts. The MV-repair rates in both accesses (PS = 65.2% vs. FS = 59.8%, *p* = 0.305) show no statistical significance. However, the rate of atrial fibrillation ablation was higher in the full sternotomy cohort (15.2% vs. 7.9%, *p* = 0.004) (Table 2).

### 3.2. Outcomes

#### 3.2.1. 30-Day Mortality and 1-Year Survival

**Figure 2:**
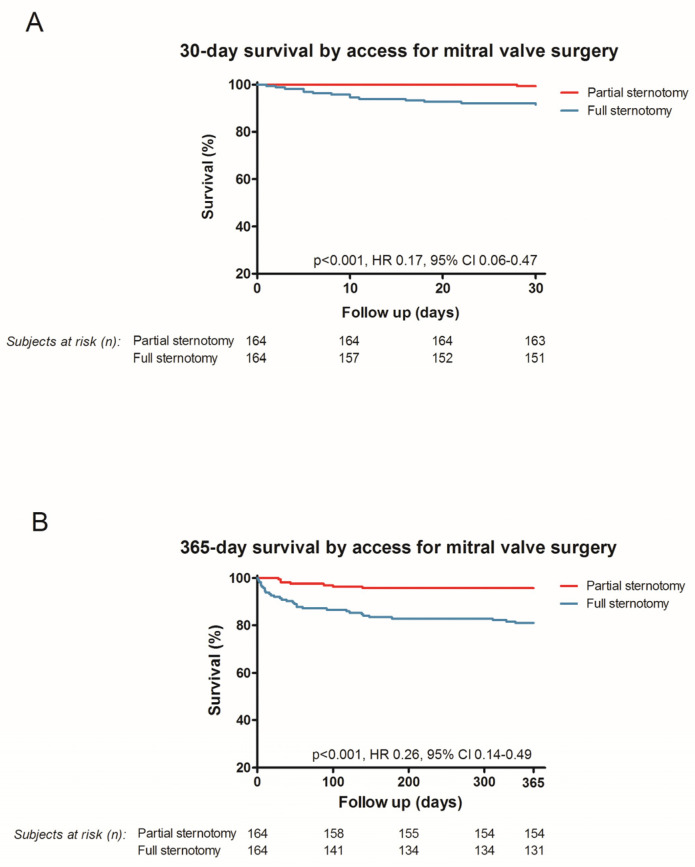
Kaplan–Meier curves for 30 days (**A**) and one year (**B**).

**Figure 3:**
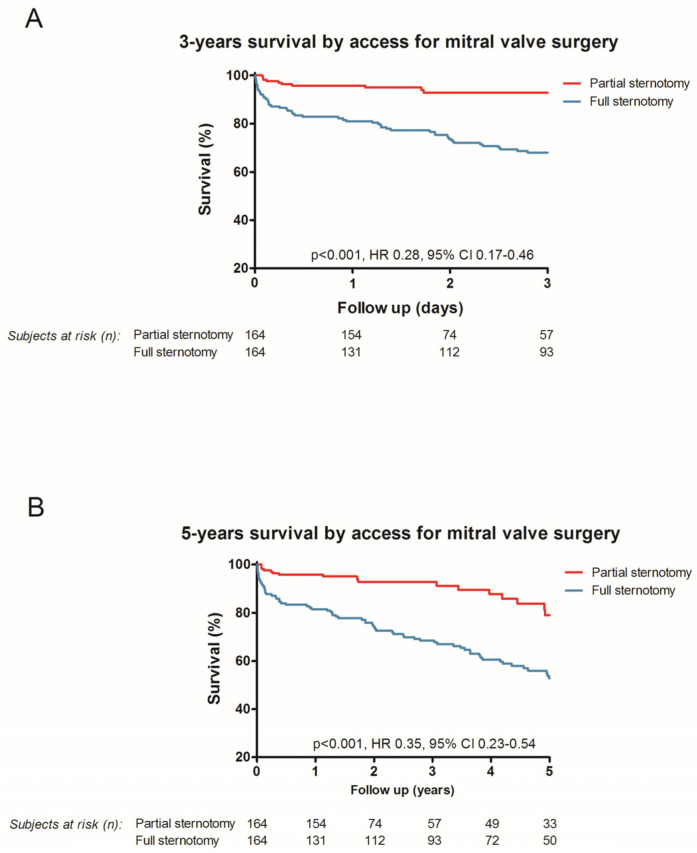
Kaplan–Meier curves for three years (**A**) and five years (**B**).

During a median follow-up time of 918 days (410–1831, upper hemi-sternotomy of 635 days (438–1652 days) and full sternotomy of 1313 days (639–2025 days)) all-cause mortality was 27.2% (89 of 328 patients) (Figure 2). During the 30-day follow up the survival in the upper hemi-sternotomy cohort was 99.4% (*n* = 163), whereas only 92.1% (*n* = 151) survived in the full sternotomy cohort (HR 0.17, 95% CI 0.06–0.47; *p* < 0.001). Analysis for one-year survival revealed a statistically significant improvement in survival when using the upper hemi-sternotomy approach (upper hemi-sternotomy 93.9%, full sternotomy 79.9%, *p* < 0.001, HR 0.26, 95% CI 0.14–0.49) as well (Figure 2). In addition, the three (HR 0.28, 95% CI 0.17–0.46; *p* < 0.001) and five (HR 0.35, 95% CI 0.23–0.54; *p* < 0.001) year survival benefits are presented in Figure 3.

#### 3.2.2. Additional Outcomes

Results are summarized in Table 3. The duration of mechanical ventilation (13 vs. 7 h, *p* < 0.001) was shorter and the prevalence of low cardiac output syndrome (31.1% vs. 18.9%, *p* = 0.011) was lower in the upper hemi-sternotomy cohort. Moreover, hospital (9 vs. 8 days, *p* < 0.001) and intensive care unit lengths of stay (2 vs. 1 days, *p* < 0.001) were significantly shorter in the upper hemi-sternotomy access compared to the full sternotomy cohort. Aortic cross-clamp and cardiopulmonary bypass times were identical in both cohorts. In addition, postoperative pacemaker implantation rates were also similar. In four patients of the upper hemi-sternotomy cohort, conversion to full sternotomy was necessary. None of the remaining postoperative outcome parameters yielded statistical significance.

## 4. Discussion

In patients with isolated or multi-valve mitral surgery, who were unsuitable for a totally endoscopic or video assisted right mini-thoracotomy approach due to contraindications, it is still uncertain if the less invasive upper hemi-sternotomy provides substantial benefit over full sternotomy. Just as most minimally invasive cardiac surgeons, we generally prefer right mini-thoracotomy for mitral valve surgery. However, in daily routines there exist numerous mitral patients with certain contraindications. For most of these patients the preferred standard access is full sternotomy and a less invasive access via upper hemi-sternotomy is not even considered by many cardiac surgeons as an alternative.

Several studies have described the non-inferiority of upper hemi-sternotomy in patients with isolated or multi-valve mitral surgery compared to full sternotomy. To avoid a selection bias, we performed propensity score matching based on all relevant anatomical and clinical conditions that may influence the access decision in order to build two comparable populations. However, this is the first investigation that describes a superiority of survival. To our knowledge, this is the first study in a sufficiently large cohort using propensity score matching that demonstrates a reduced mortality in the upper hemi-sternotomy cohort compared to full sternotomy within one-year follow up. The mortality rate in the upper hemi-sternotomy group in our investigation is lower than in earlier publications using this less invasive access [11,12,20]. It is noteworthy that only one patient in the upper hemi-sternotomy group died within 30 days, whereas 30-day mortality in the full sternotomy group was 7.9% (*n* = 13). The one-year mortality with comparable preoperative EuroSCORE II scores is similar with the recently published study of 419 patients with this access [21]. However, both results are almost 3-fold higher than by an additional investigation by Svensson et al. [22]. One possible reason might be that in this study even patients with low risk profile, who were candidates for mini-thoracotomy-MVS, were predominantly operated via upper or lower hemi-sternotomy. We believe that mini-thoracotomy for MVS should be the preferred access and only patients with contraindications or higher risk profiles should be operated via upper hemi-sternotomy. This negative selection might explain the higher mortality rates in our center. Nevertheless, even in complex mitral valve pathologies, similarly to mitral annular calcifications, the mortality rates are comparable [23]. However, the one-year mortality rate in this investigation is higher (6.1%) than the previously presented data of our center with 3.3% [13]. The higher mortality can be explained by the fact that with growing experience in recent years, more complex patients were operated on via this access.

Before this investigation, our surgeons assumed that in the PS-MVS cohort, patients were healthier, the extent of mitral annulus calcifications was smaller and the rates of MV replacement and prevalence of isolated MVS were higher than in the FS group. Therefore, these parameters were used as criteria for selection of the right access. Surprisingly, our data did not support this assumption. However, only pulmonary hypertension was higher in the FS-MVS cohort after propensity score matching, whereas the incidence of acute cardiac decompensation was significantly higher in the PS group.

**Figure 4:**
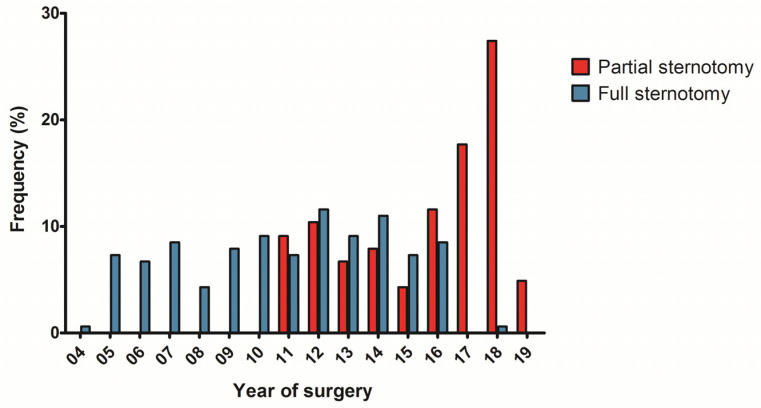
Distribution of access in the year of surgery after propensity score matching [12].

Mini-thoracotomy has been the preferred access for mitral valve surgery in our center since 2001. This strategy results in the fact that these are, in general, at a lower risk and only patients with a certain risk profile formed our full or hemi-sternotomy study cohorts. It has to be pointed out that since implementation of upper hemi-sternotomy only 91 patients of the study cohort were operated via complete sternotomy (Figure 4).

Perhaps one of the main reasons for the skepticism against the hemi-sternotomy access, which initially also existed in our department, is the assumption that the limited surgical exposure may result in longer cardiopulmonary bypass and aortic cross-clamp times. Consequently, higher rates of complications would be expected. Our data indicate that operative times were not different for both approaches. In addition, mitral valve repair rates and the prevalence of moderate to severe mitral annulus calcification were also comparable, indicating excellent exposure and access to the mitral valve. We have learned that a major contribution to improve surgical exposure is percutaneous cannulation of the femoral vein instead of direct cannulation of the inferior vena cava, which previously has not been described in the published literature.

In our series the conversion rate to full sternotomy was 6.56% (*n* = 4). In only one patient limited exposure of the mitral valve forced us to convert to full sternotomy. In any case, exposure of mitral valve was extremely difficult even thereafter. In one patient, atrioventricular dehiscence after following en-bloque decalcification and repair of the mitral valve annulus occurred and could be managed only by full sternotomy. Further reasons for conversion were pericarditis with severe adhesions (*n* = 1) and post cardiopulmonary bypass myocardial ischemia (*n* = 1). It has to be pointed out that the advantage of this less invasive access is the straightforward and safe possibility of conversion to full sternotomy at any time.

Another argument against upper hemi-sternotomy is that the extended transseptal incision is accused of a greater risk of sinus node dysfunction due to injury to the sinus node artery. This access related complication was regarded as the main cause of postoperative permanent pacemaker implantation [24]. Our data indicates similar rates of pacemaker implantation when using the extended transseptal compared to the full sternotomy interatrial groove approach (3.7% vs. 5.5%).

Indications for upper hemi-sternotomy were evolving over the years with growing experience. In the first years after implementation of this access we learnt to operate acute endocarditis, perform additional ascending aortic surgery and even selected coronary bypass grafting. With atrial ablation surgery we were extremely reserved in the initial years but with growing expertise we are now also routinely providing atrial fibrillation ablation with this approach. This fact also explains the higher rate of atrial fibrillation surgery in the full sternotomy cohort. Major annulus calcification is an absolute contraindication for mini-thoracotomy due to no dedicated long-shafted instruments. Upper hemi-sternotomy, in contrast, also allows en-bloque decalcification and annulus reconstruction with a less invasive approach in these patients, allowing this access in almost all MVS candidates. However, primary indications for full sternotomy are still re-do cases, complex myocardial revascularizations with or without the use of arterial grafts and dilatative cardiomyopathy with a high risk of possible implanting mechanical circulatory support systems.

Further analysis of the results revealed lower incidences of low cardiac output syndrome in the upper hemi-sternotomy cohort. There is evidence that the partial integrity of the pericardium protects against perioperative right heart failure due to prevention of right ventricular dilatation, resulting in low cardiac output syndrome [25,26]. However, the routine insertion and liberal use of temporary AV-pacemaker wires may have also played a role in the prevention of right heart failure. Moreover, upper hemi-sternotomy reduces sternal trauma and better preserves the thoracic integrity especially in the diaphragmal part, and consequently in a more adequate respiratory function and better patient compliance with physiotherapy [27]. All these factors might have led to the reduced duration of mechanical ventilation and length of intensive care unit and hospital stay, and would therefore stand in agreement with some investigations of aortic valve surgery via upper hemi-sternotomy [28,29,30,31]. These outcomes were also observed in our investigation. However, there might be different dynamics of the incision length of pericardiotomy in aortic and mitral valve disease. Nevertheless, it must be pointed out that all these factors may have an impact on short-term mortality, but the possible reasons for increased mid- or long-term survival remain unclear. Our data reveal the highest mortality rates within the first year. This might be one of the possible reasons why this advantage shows the following statistical significances in later timepoints in favor of increased survival in the upper hemi-sternotomy cohort.

In fact, one of the crucial aspects of our investigation that the preoperative data revealed is that the patients in both cohorts are similar to each other.

Finally, upper hemi-sternotomy mitral valve surgery can be integrated into surgical training comparable to the full median sternotomy approach.

## 5. Limitations

One major limitation of this investigation is the adjudication of patients to one or the other approach. With continuous evolution of technique and growing experience this changed dramatically. While in the first era of the study period (2004–2010) all patients who were regarded as unsuitable for mini-thoracotomy were operated through full sternotomy, since 2011 an increasing proportion were subjected to partial sternotomy, now leaving only rare indications for full sternotomy. With certain exclusion criteria (urgent surgery, endocarditis, re-operations, ascending aorta surgery, CABG, etc.), we tried to create two homogenous cohorts to compare. To further reduce differences in baseline risk factors, propensity score matching creating to comparable cohorts was applied. It is clear that this statistical technique is not the ideal way of analysis for different patient populations and might be criticized. Nevertheless, it is the best possible method to create homogenous cohorts for comparison of two procedures [32]. Furthermore, decision making as to full or upper hemi-sternotomy cannot be clearly defined, as all surgeons (senior and junior) prefer upper hemi-sternotomy access because conversion to full sternotomy is possible at any time, which might be a major bias.

Further limitations were the retrospective data analysis and the relatively small sample size. Therefore, we cannot definitively rule out that the study was underpowered for the assessment of differences between study groups with regard to some outcome parameters.

## 6. Conclusions

The presented results demonstrate that upper hemi-sternotomy is a complementary and less invasive approach, which can close the gap between minimally invasive mini-thoracotomy and the necessity for full sternotomy in mitral valve surgery. For patients without a prohibitive risk profile, endoscopic mitral valve surgery with 3D imaging through right mini-thoracotomy is our preferred approach. In addition, we have adapted the less invasive hemi-sternotomy as a standard approach in patients with a certain risk profile instead of full sternotomy.

## Figures and Tables

**Table 1:** Baseline characteristics of the study cohorts before and after propensity score matching.

	Unmatched Data			After Propensity Score-Matching		
	Full Sternotomy *n* = 350	Upper Hemi-Sternotomy *n* = 167	*p*-Value	Full Sternotomy *n* = 164	Upper Hemi-Sternotomy *n* = 164	*p*-Value
Age (years) ^1^	69 (60–74)	71 (63–77)	0.027	72 (64–76)	71 (63–77)	0.859
Gender, males (%, *n*)	47.7 (167)	44.3 (74)	0.468	50 (82)	43.3 (71)	0.223
Body surface area (m^2^)	1.81 (1.66–1.96)	1.81 (1.65–1.93)	0.323	1.79 (1.65–1.94)	1.81 (1.65–1.93)	0.830
Diabetes Mellitus (%, *n*)	12 (42)	18.3 (18)	0.055	12.2 (20)	18.3 (30)	0.125
Arterial hypertension (%, *n*)	64.2 (224)	85.9 (85)	<0.001	68.3 (112)	81.1 (133)	0.008
COPD (%, *n*)	35.4 (124)	30.5 (51)	0.292	35.4 (58)	30.5 (50)	0.347
PAOD (%, *n*)	2.3 (8)	4.2 (7)	0.223	2.4 (4)	4.3 (7)	0.358
Dialysis (%, *n*)	0.9 (3)	3 (5)	0.064	0.6 (1)	3.0 (5)	0.099
Prev.CVE (%, *n*)	4.3 (15)	4.8 (8)	0.762	6.7 (11)	4.9 (8)	0.478
Acute HF < 3 months (%, *n*)	4 (14)	12.6 (21)	<0.001	5.5 (9)	12.8 (21)	0.022
EuroSCORE II (%) ^1^	3.04 (1.69–4.84)	3.0 (1.67–4.74)	0.874	3.14 (1.88–4.95)	3.0 (1.67–4.74)	0.267
LV-ejection fraction (%) ^1^	55 (47–61)	59 (50–65)	0.005	57 (49–63)	59 (50–65)	0.659
NYHA III (%, *n*)	54.0 (189)	59.3 (99)	0.228	56.1 (92)	59.8 (98)	0.502
NYHA IV (%, *n*)	5.7 (20)	5.4 (9)	0.893	5.5 (9)	5.5 (9)	n.a
Interm.-atrial fibrillation (%, *n*)	46.9 (164)	37.1 (62)	0.054	43.9 (72)	37.8 (62)	0.261
Perm.-atrial fibrillation (%, *n*)	23.2 (81)	17.4 (29)	0.159	22.0 (36)	17.7 (29)	0.332
sPAP (mmHg) ^1^	50 (36–60)	45 (36–57)	0.135	50 (40–60)	45 (36–57)	0.024
NT-proBNP (ng/L) ^1^	1189 (619–2498)	1080 (414–2535)	0.323	1248 (618–2476)	1080 (430–2451)	0.238

Abbreviations: COPD = chronic obstructive pulmonary disease; CVE = cerebrovascular event; HF = heart failure; PAOD = peripheral arterial occlusive disease; sPAP = systolic pulmonary artery pressure. ^1^ Continuous variables are expressed as median and interquartile range.

**Table 2:** Intraoperative parameters after propensity score matching.

	Propensity Score Matched		
	Full Sternotomy *n* = 164	Upper Hemi-Sternotomy *n* = 164	*p*-Value
MV-etiology			
Primary (%, *n*)	87.7 (144)	86.0 (141)	0.624
Secondary (%, *n*)	12.2 (20)	14.0 (23)	0.624
Moderate to severe AC (%, *n*)	35.4 (58)	36.0 (59)	0.908
Surgical interventions			
MVS isolated (%, *n*)	47.6 (78)	50.6 (83)	0.581
MV repair (%, *n*)	59.8 (98)	65.2 (107)	0.305
MV + TV-surgery (%, *n*)	30.5 (50)	31.1 (51)	0.905
MV + AV-surgery (%, *n*)	18.3 (30)	15.9 (26)	0.557
MV + AV + TV-surgery (%, *n*)	3.7 (6)	2.4 (4)	0.521
Atrial fibrillation surgery (%, *n*)	15.2 (25)	7.9 (13)	0.038

AC = annulus calcification; AV = aortic valve; MV = mitral valve; MVS = mitral valve surgery; TV = tricuspid valve.

**Table 3:** Secondary intra- and postoperative endpoints in the propensity score matched cohorts.

	Full Sternotomy *n* = 164	Upper Hemi-Sternotomy *n* = 164	*p*-Value
CPB time (min) ^1^	161 (130–196)	164 (140–196)	0.262
Aortic X-clamp time (min) ^1^	107 (81–133)	106 (88–132)	0.829
Second pump run/X-clamp (%, *n*)	9.8 (16)	6.1 (10)	0.220
ECMO (%, *n*)	2.4 (4)	1.2 (2)	0.545
LOS (%, *n*)	31.1 (51)	18.9 (31)	0.011
Tamponade or excessive bleeding (%, *n*)	4.3 (7)	3.0 (5)	0.556
Hemofiltration/-dialysis new (%, *n*)	17.1 (28)	12.8 (21)	0.278
Ventilation length (h) ^1^	13 (7–30)	8 (5–17)	<0.001
Red blood units (first 24 h) ^1^	1 (0–3)	1 (0–3)	n.a.
ICU length (days) ^1^	2 (1–14)	1 (1–3)	<0.001
Hospital stay (days) ^1^	9 (8–13)	8 (7–10)	<0.001
MOF (%, *n*)	6.7 (11)	4.3 (7)	0.332
Sepsis (%, *n*)	5.5 (9)	2.4 (4)	0.157
Pneumonia (%, *n*)	4.9 (8)	3.0 (5)	0.396
Deep wound infection (%, *n*)	3.7 (6)	3.0 (5)	0.759
Stroke (%, *n*)	3.0 (5)	1.2 (2)	0.252
PM-implantation (%, *n*)	5.5 (9)	3.7 (6)	0.428

Aortic X-clamp-time = aortic cross-clamp time; CPB time = cardiopulmonary by-pass time; ECMO = extracorporeal membrane oxygenation; ICU = intensive care unit; LOS = cardiac low output syndrome; MOF = multi organ failure; PM = pacemaker.^1^ Continuous variables are expressed as median and interquartile range.

## Data Availability

Clinical data were obtained from the database of our mitral valve surgery program and from the hospital’s digital patient management system.
